# *In vitro* evaluation of antibacterial activity of verbascoside, lemon verbena extract and caffeine in combination with gentamicin against drug-resistant *Staphylococcus aureus* and *Escherichia coli* clinical isolates

**Published:** 2018

**Authors:** Bibi Sedigheh Fazly Bazzaz, Bahman Khameneh, Mohammad Reza Zahedian Ostad, Hossein Hosseinzadeh

**Affiliations:** 1 *Biotechnology Research Center, Pharmaceutical Technology Institute, Mashhad University of Medical Sciences, Mashhad, Iran *; 2 *Department of Pharmaceutical Control, School of Pharmacy, Mashhad University of Medical Sciences, * *‎* *Mashhad, Iran*; 3 *Students Research Committee, School of Pharmacy, Mashhad University of Medical Sciences, Mashhad, Iran*; 4 *Pharmaceutical Research Center, Pharmaceutical Research Center,* *Pharmaceutical Technology Institute,* *Mashhad University of Medical Sciences,* *Mashhad, Iran, Mashhad University of Medical Sciences, Mashhad, Iran*

**Keywords:** Antibiotics, Combination therapy, Methylxanthines‎, Herbal extract, Natural compounds, Resistant pathogens

## Abstract

**Objective:**

In recent years, there has been an increasing interest in using herbal products to overcome bacterial resistance. The aim of this study was to investigate the effect of lemon verbena aqueous extract, verbascoside and caffeine in combination with gentamicin against standard and clinical isolates of *Staphylococcus aureus* and *Escherichia coli* strains.

**Materials and Methods:**

The MIC and MBC values of different antibacterial agents against bacterial strains were determined. The effect of co-administration lemon verbena extract, verbascoside, and caffeine and gentamicin was studied *in vitro* using a checkerboard method and calculating fraction inhibitory concentration index (FICI).

**Results:**

Herbal extract, verbascoside and caffeine alone showed no inhibitory effects on any of the bacterial strains (at doses up to 200 g/ml). Herbal extract, verbascoside and caffeine were able to decrease the MIC of gentamicin against the standard resistant strains and two clinical isolates. Among these combinations, the co-administration of verbascoside and gentamicin was more effective and synergistic activities (FICI<1)‏ against clinical isolates were observed.‏

**Conclusion:**

The results of the present study revealed that herbal extract, verbascoside and caffeine potentiated the antimicrobial action of gentamicin against some clinical isolates of *S. aureus* and *E. coli*.

## Introduction

Anti-bacterial resistance is one of the greatest challenges of the twenty-first century (Rasko and Sperandio, 2010 ▶). Infectious diseases caused by resistant strains have numerous negative effects as their treatment requires higher doses of antibiotics, additional treatments, and prolonged hospitalizations and causes higher mortality rates (Khameneh et al., 2016 ▶). 

Recently, many strategies have been proposed to combat bacterial resistance. Developing novel structural and functional classes of antibiotics, combination therapy, applying natural compounds and using novel drug delivery systems are the main approaches in this field (Khameneh et al., 2015 ▶). 

Combination therapy has several potential advantages including enhancing the antibiotic activity, preventing the emergence of resistance and reducing the risk of infection (Hagihara et al., 2012 ▶). Therefore, various compounds have been tested in combination with antibiotics. These days, the investigators' attention is increasingly attracted to natural compounds which have shown potent antibacterial activities when used alone or in combination with other antibacterial agents (Akaberi et al., 2015 ▶, Forouzanfar et al., 2014 ▶).

*Lippia citriodora* (lemon verbena) has been widely used in traditional medicine for its pharmacological properties such as antispasmodic, antipyretic, sedative, and digestive activities (Carnat et al., 1999 ▶, Quirantes-Pine et al., 2013 ▶). *L. citriodora* leaves contain many polar compounds like phenyl propanoids, flavonoids, phenolic acids and iridoid glycosides (Quirantes-Pine et al., 2013 ▶). 

Verbascoside has been mainly found in the *Verbascum* species but has also been detected in more than 200 plant species (Alipieva et al., 2014 ▶). This compound is the major substance in lemon verbena and has several properties such as anti-inflammatory, antioxidant, antitumor and antimicrobial activities (Quirantes-Pine et al., 2013 ▶). Verbascoside is a member of the phenylpropanoid glycosides family which is structurally characterized by the caffeic acid moiety and 4,5-hydroxyphenylethanol (hydroxytyrosol) bound to a -(d)- glucopyranoside, through ester and glycosidic links, respectively, with a rhamnose in sequence (1–3) to the glucose molecule (Singh et al., 2010 ▶). 

The antibacterial activities of lemon verbena extract and verbascoside have received a great deal of attention (Ghaemi et al., 2007 ▶). The ability of plants rich in verbascoside for treatment of microbial infections, has been previously described (Georgiev et al., 2012 ▶). It has been reported that verbascoside can be a promising therapeutic agent for treatment of microbial infections. In another study, the efficacy of verbascoside for treatment of acne vulgaris was mentioned (Azimi et al., 2012 ▶).

The mechanism of antibacterial action of verbascoside is not fully understood but the capability of verbascoside to modulate membrane-dependent cellular processes might be involved in its antibacterial action (Funes et al., 2010 ▶). 

Combination of chemical compounds with antibacterial agents is another effective approach. Methylxanthines are potent bronchodilators used to treat acute asthma. Moreover, there is some evidence that these compounds show antibacterial properties against some bacterial pathogens (Elgaher et al., 2009 ▶, Hayallah et al., 2011 ▶). Aminophylline and caffeine, for instance, increased the antimicrobial action of carbenicillin, ceftizoxime and gentamicin against *Staphylococcus aureus* and *Pseudomonas aeruginosa* (Hosseinzadeh et al., 2006 ▶). Also, caffeine has shown antibacterial properties along with potent antifungal activity against *Candida albicans *(Gyawali et al., 2014 ▶, Kim et al., 2013 ▶). 

Gentamicin is a potent antibiotic which has been used for the treatment of a wide range of infections caused by Gram-positive and Gram-negative bacteria (Corvec et al., 2013 ▶, Pantosti et al., 2007 ▶). Despite all advantages, this antibiotic suffers from some shortcomings such as undesirable side effects and increased microbial resistance. In this regard, combination of gentamicin with other antibacterial agents may reduce the risk of increasing resistance and toxicity. 

The aim of the present study was to evaluate the antibacterial activities of caffeine, lemon verbena extract and verbascoside alone and in combination with gentamicin against two clinically important bacteria namely, *Escherichia coli *and *S. aureus*. 

## Materials and Methods

**Materials**

*L. citriodora* leaves were collected from the surrounding areas of Karaj city, Alborz province, Iran, dried in shadow and grounded to powder. *L. citriodora* was identified by the Department of Botany Ferdowsi University (Mashhad, Iran), and voucher samples were preserved in the Herbarium of the Department of Pharmacognosy, School of Pharmacy, Mashhad University of Medical Sciences, Mashhad, Iran. Verbascoside was purchased from Extrasynthese (Genay, France). Caffeine and gentamicin were obtained from Hakim Pharmaceutical Company (Tehran, Iran).

**Preparation of** ***L. citriodora*** **aqueous extract**

The aqueous extract of *L. citriodora* was prepared by adding 1 L of distilled water to 100 g of powdered plant material in a 2.5 L glass flask and boiled for 15 min. The solution was subsequently filtered using Whatman No. 1 filter paper and the obtained residue was stored in a freezer at −70°C and then freeze-dried.

**Bacterial strains**

The microorganisms used in this study were *S. aureus* (five strains including one methicillin-resistant strain [MRSA ATCC 43300] and four isolated strains) and *E. coli* (two isolated strains). *S. aureus* were isolated from acne (two strains), eye and urinary tract infections.

**Determination of minimum inhibitory and minimum bactericidal concentrations of antibacterial agents**

The minimum inhibitory concentration (MIC) of extracts and compounds against bacteria was determined as previously described (Khameneh et al., 2015 ▶). Briefly, approximately 10^6^ CFU/ml cells from overnight cultures were used as inoculum. Serial dilutions of each tested compound were prepared in Muller Hinton Broth (MHB) (Difco) in 96-well microtiter plates. Then, the inoculum was added to each well to obtain a final bacterial concentration of 10^5^ CFU/ml. The inoculated microplates were incubated at 37 ^°^C for 24 hr under aerobic condition. MIC was determined by adding TTC (triphenyl tetrazolium chloride, Merck) to each well at a concentration of 0.05% followed by incubation at 37 ^°^C for 30 min. MICs were defined as the lowest concentration of tested compound that did not reduce TTC to red formazan.

For MBC determination, an aliquot of 10 μl from all wells with no visible growth (no red color), was seeded in Tryptone Soya Agar plates (TSA) (Lab M; Bury, UK). The plates were then incubated at 37^°^C, overnight. MBC is defined as the lowest concentration of antimicrobial agent that kills >99.9% of bacteria.

**Evaluation of synergistic effect**

To evaluate the antibacterial activities of two antibacterial agents, the checkerboard method was used. In brief, serial 2-fold dilutions of gentamicin and other antimicrobial agents were mixed in each well of a 96-well microtiter plate (250 µL). So, each row and column contained a constant amount of one antibacterial agent and increasing amounts of the second agent. The MIC was assessed as mentioned above and finally the Fractional Inhibitory Concentration Index (FICI) value was used to assess whether synergism, indifference or antagonism occurred following the co-administration of the two evaluated antibacterial agents (Khameneh et al., 2015 ▶). 

The FICI of the antibacterial agent combination was FIC of drug A + FIC of drug B, where the FIC of drug A= (MIC of drug A in combination)/(MIC of drug A alone) and the FIC of drug B= (MIC of drug B in combination)/(MIC of drug B alone). The combination effects were evaluated based on the following criteria: FICI<0.5 denoting synergy; 0.5<FICI >0.75 denoting partial synergy; 0.76< FICI <1 denoting an additive effect; 1< FICI <4 denoting indifference; and FICI >4 denoting antagonism (Khameneh et al., 2015 ▶).

**Statistical analysis**

All tests were performed at least in triplicate. A one-way analysis of variance (ANOVA) was used for testing overall group differences. Differences between mean were statistically considered significant if the p value was less than 0.05.

## Results

**Determination of MIC and MBC**

The MIC and MBC values of gentamicin for each clinical isolate or reference bacteria were noted in Table 1. MIC and MBC of other tested compounds were higher than 200 μg/ml.

**Table 1:** MIC values of gentamicin against 7 clinically resistant *Staphylococcus aureus *and* Escherichia coli ‎* strains.

	Strain	Gentamicin	
		MIC/MBC (μg/ml)	
*Staphylococcus aureus*	1	>80	>80
*Staphylococcus aureus*	2	20	>80
*Staphylococcus aureus*	3	>80	>80
*Staphylococcus aureus*	4	>80	>80
*Staphylococcus aureus * (MRSA ATCC 43300)	5	50	>80
*Escherichia coli * *‎*	1	>80	>80
*Escherichia coli * *‎*	2	50	>80

**Evaluation of synergistic effect**

The combined effects of gentamicin and the different compounds were shown in Tables 2-4. 

These results indicated that combination of gentamicin and caffeine was not effective against bacterial strains except for only one clinical isolate (*S. aureus *No.2*).*

According to the results (Tables 3 and 4), it was concluded that combination of gentamicin with natural compounds, results in partial synergistic effects. For instance, combination of gentamicin with lemon ‎verbena ‎extract was effective against some strains of *E. coli* and *S. aureus*. It should be noted that combination of gentamicin with natural product such as verbascoside was more effective in comparison with mono-antibiotic therapy. 

**Table 2:** Results of application of a combination of gentamicin and caffeine against *Staphylococcus aureus *and* Escherichia coli.*

Bacterial strains	Strain	Agent	MIC (μg/ml)		FIC	FICI	Outcome
			Alone	gentamicin+ caffeine			
*Staphylococcus aureus*	1	gentamicin	>80	>80	1	2	Indifference
		caffeine	>200	>80	1		
*Staphylococcus aureus*	2	gentamicin	12.5	6.25	0.5	0.56	Partial synergy
		caffeine	>200	12.5	0.06		
*Staphylococcus aureus*	3	gentamicin	>80	>80	1	2	Indifference
		caffeine	>200	>80	1		
*Staphylococcus aureus*	4	gentamicin	>80	>80	1	2	Indifference
		caffeine	>200	>80	1		
*Staphylococcus aureus *(MRSA ATCC 43300)	5	gentamicin	50	25	0.5	1.5	Indifference
		caffeine	>200	200	1		
*Escherichia coli*	1	gentamicin	>80	80	1	1.25	Indifference
		caffeine	>200	50	0.25		
*Escherichia coli*	2	gentamicin	50	50	1	2	Indifference
		caffeine	>200	200	1		

**Table 3:** Results of application of a combination of gentamicin and lemon verbena extract against *Staphylococcus aureus *and* Escherichia coli*

Bacterial strains	Strain	Agent	MIC (μg/ml)		FIC	FICI	Outcome
			Alone	gentamicin + herbal extract			
*Staphylococcus aureus*	1	gentamicin	>80	>80	1	2	Indifference
		lemon verbena extract	>200	>80	1		
*Staphylococcus aureus*	2	gentamicin	12.5	6.25	0.5	0.51	Partial synergy
		lemon verbena extract	>200	3.1	0.06		
*Staphylococcus aureus*	3	gentamicin	>80	>80	1	2	Indifference
		lemon verbena extract	>200	>80	1		
*Staphylococcus aureus*	4	gentamicin	>80	>80	1	2	Indifference
		lemon verbena extract	>200	>80	1		
*Staphylococcus aureus *(MRSA ATCC 43300)	5	gentamicin	50	50	1	2	Indifference
		lemon verbena extract	>200	200	1		
*Escherichia coli*	1	gentamicin	>80	80	1	2	Indifference
		lemon verbena extract	>200	200	1		
*Escherichia coli*	2	gentamicin	50	25	0.5	0.75	Partial synergy
		lemon verbena extract	>200	50	0.25		

**Table 4:** Results of application of a combination of gentamicin and verbascoside against *Staphylococcus aureus *and* Escherichia coli.*

Bacterial strains	Strain	Agent	MIC (μg/ml)		FIC	FICI	Outcome
			Alone	gentamicin + verbascoside			
*Staphylococcus aureus*	1	gentamicin	>80	>80	1	2	Indifference
		verbascoside	>200	>80	1		
*Staphylococcus aureus*	2	gentamicin	12.5	6.25	0.5	0.75	Partial synergy
		verbascoside	>200	50	0.25		
*Staphylococcus aureus*	3	gentamicin	>80	>80	1	2	Indifference
		verbascoside	>200	>80	1		
*Staphylococcus aureus*	4	gentamicin	>80	>80	1	2	Indifference
		verbascoside	>200	>80	1		
*Staphylococcus aureus *(MRSA ATCC 43300)	5	gentamicin	50	25	0.5	1	Additive
		verbascoside	>200	100	0.5		
*Escherichia coli*	1	gentamicin	>80	80	1	1.25	Indifference
		*lemon verbena* extract	>200	25	0.25		
*Escherichia coli*	2	gentamicin	50	25	0.5	0.53	Partial synergy
		*lemon verbena* extract	>200	6.25	0.03		

## Discussion

Clinically-isolated bacteria are regarded as main causes of nosocomial infections. These types of infections pose serious threats to the public health and a cause wide range of problems. So, finding a proper solution seems to be important. Combination therapy is an effective approach to reduce the risk of nosocomial infection which is currently attracting marked attention. Therefore, in the present study, different combinations of gentamicin and some therapeutic agents were evaluated.

The MIC and MBC values of gentamicin against bacterial species (Table 1) indicated that with respect to the standard strain (MRSA ATCC 43300), isolated species were more resistant to antimicrobial agents. These evidence reflected that the bacterial species used in this study, showed high levels of resistance to antibacterial agents. To enhance the antibacterial activity, three compounds were used in combination with gentamicin.

Methylxanthines are useful therapeutic agents administered for treatment of acute asthma. These agents were also used for treatment of bacterial infections (Bazzaz et al., 2012 ▶, Hosseinzadeh et al., 2006 ▶). In the present study, the combinatorial effect of caffeine and gentamicin against bacterial species were investigated (Table 2). According to our data, the synergistic effects were only observed against one clinical isolate of *S. aureus*. These findings supported that caffeine might be more efficient against Gram-positive bacteria compared to Gram-negative ones. These results were in line with previously published data which showed that some derivatives of methylxanthines exert antibacterial activates especially against Gram-positive species (Elgaher et al., 2009 ▶). Additionally, it was shown that concomitant intake of the antibiotics and caffeine potentiated the antibacterial effect of antibiotics against *S. aureus* (Esimone et al., 2008 ▶). 

These evidence support the hypothesis that extracts with high levels of verbascoside can show the antibacterial activities. In previous studies, the antibacterial properties of A*rrabidaea harleyi* A.H. Gentry extracts containing verbascoside, were proven (Lima et al., 2003 ▶). Also, it was shown that herbal extracts which contain flavonoids, alkaloids, tannins and phenolic compounds, affect the efflux system of bacteria (Coutinho et al., 2009 ▶). These findings were consistent with our data (Table 3). The combination of gentamicin with lemon verba extract enhanced antibacterial activities of antibiotics. These results support some ethnopharmacological uses of this plant (Lima et al., 2003 ▶).

*Buddleja globosa* leaves showed antibacterial activity against *S. aureus* and *E. coli*. Based on the antimicrobial activity, the extract was fractionated and verbascoside was isolated (Pardo et al., 1993 ▶)‎. Verbascoside belongs to the phenylpropanoid glycosides family with demonstrate anti-inflammatory, antioxidant, antitumor and antimicrobial properties (Alipieva et al., 2014 ▶, Pardo et al., 1993 ▶). As seen in Table 4, upon combination of gentamicin with verbascoside, partial synergistic activities were observed in some clinical isolates of *S. aureus* and *E. coli*. In case of standard strain, additive effects were seen. Additionally, in comparison with lemon verba extract, the antibacterial activity of verbascoside was more pronounced. These data indicated that pure compounds such as verbascoside showed better activity than herbal extracts. These findings were also in line with previous findings which showed that verbascoside has antibacterial activities (Alipieva et al., 2014 ▶). The antibacterial action of verbascoside was not fully understood, but it was suggested that it is related to inhibition of leucine uptake and bacterial protein synthesis (Guillermo Avila et al., 1999 ▶). It was demonstrated that verbascoside was more effective against Gram-positive bacteria and this effect was due to perturbing the phospholipid/water interface of membranes and consequently increasing the surface area of the phospholipid head groups in the bilayers (Funes et al., 2010 ▶).

The results of this *in vitro* study highlighted the advantages of antibiotic combination with natural and chemical compounds. This approach can be used as a promising solution for combating bacterial infections. 

## Conflict of interest

The authors have no conflict of interests to declare.
